# Fabrication and Performance Evaluation of 3D-Printed Zinc–Manganese Flexible Batteries

**DOI:** 10.3390/ma19071309

**Published:** 2026-03-26

**Authors:** Ernan Ju, Cong Yan, Li Wu

**Affiliations:** 1 School of Intelligence & Electronic Engineering, Dalian Neusoft University of Information, Dalian 116025, China; juernan@neusoft.edu.cn; 2 School of Mechanical Engineering, Dalian Jiaotong University, Dalian 116028, China; yancong138537@163.com

**Keywords:** 3D printing, MnO_2_/acetylene black cathode, zinc-manganese flexible battery, mechanical properties, electrochemical performance

## Abstract

To meet the requirements of flexibility and high performance for energy storage devices in flexible wearable electronic equipment, the MnO_2_/acetylene black composite flexible cathodes is fabricated via 3D printing technology and the aqueous manganese-based zinc-ion flexible batteries are assembled. Based on bending and torsion mechanical tests, and the electrochemical tests, the optimal 3D printing electrode structure was determined. The micromorphology of the electrode after mechanical tests shows that when the printed lines of the upper and lower layers form a 30° angle, the electrode sheet exhibits the least damage. Electrochemical tests indicated that it had an ohmic resistance of 2.052 Ω, an interfacial charge transfer resistance of 141.1 Ω, a specific capacity of 103 mAh/g at 50 mA/g, and a specific capacity of 65 mAh/g at 500 mA/g. Compared with traditional coated electrodes, the 3D-printed electrode showed significantly improved diffusion coefficient, conductivity, and cycle stability. The assembled 3D-printed flexible battery could stably power a 1.5 V LED bulb under flat, bent, and twisted states. It provides a feasible solution for the development of high-performance flexible energy storage devices.

## 1. Introduction

With the intensification of the global energy crisis and the rapid development of wearable electronic technology, the development of energy storage devices with high safety, excellent flexibility, and stable electrochemical performance has become a research hotspot in the energy field [1]. Excessive consumption of traditional fossil energy has caused serious environmental problems. Although lithium-ion batteries dominate the market of portable electronic devices, they have drawbacks such as high cost, insufficient safety, and poor environmental compatibility [2], making it difficult to meet the requirements of flexible wearable devices for lightweight, deformable, and high-safety power sources [3]. Flexible wearable electronic products have been widely used in sports monitoring, medical health, and other fields [4], but their power supply still relies on traditional rigid batteries, which greatly limits the flexibility and endurance of the devices [5]. Therefore, the development of flexible power supply devices capable of withstanding mechanical deformations such as bending and twisting is imminent [6].

3D printing technology, with its unique advantage of layer-by-layer forming, provides an innovative approach for the precise structural regulation of flexible electrodes [7]. This technology can flexibly construct complex porous structures, effectively increasing the specific surface area of electrodes, shortening ion transport paths, and optimizing the mechanical flexibility of electrodes [3]. Compared with traditional processes such as coating and vacuum filtration, it shows significant advantages in structural design freedom and synergistic performance improvement [8]. Direct Ink Writing (DIW), as one of the mainstream 3D printing technologies, has become the preferred scheme for the preparation of flexible energy storage electrodes due to its wide range of raw material choices and simple operation [9]. It can achieve stable forming of high-precision complex structures by regulating the rheological properties of inks [10].

Among various energy storage systems, manganese-based aqueous zinc-ion batteries have become an ideal choice for flexible energy storage devices due to their abundant zinc resources, low redox potential, high safety, and environmental friendliness [11]. As a typical cathode material, MnO_2_ has high theoretical specific capacity and redox activity, but its practical application is limited by problems such as poor conductivity and large volume change during cycling [12]. Acetylene black, as a high-quality conductive agent, can effectively improve the conductivity of electrodes [13]. Meanwhile, the construction of reasonable porous structures via 3D printing technology can further alleviate the inherent defects of MnO_2_ and improve the comprehensive performance of electrodes [14]. At present, some progress has been made in the application of 3D printing technology in the preparation of flexible electrodes. For example, Bao et al. prepared highly deformable electrodes via 3D printing, which exhibited excellent mechanical flexibility [7]. Ma et al. significantly improved the capacity and stability of zinc-ion batteries by constructing honeycomb-like hierarchical porous cathodes [14]. However, systematic research on the structural design, process optimization, and synergistic performance improvement of MnO_2_/acetylene black composite electrodes still needs to be further carried out.

The energy storage mechanism of aqueous zinc-ion batteries is complex, mainly including Zn^2+^ intercalation/deintercalation, H^+^/Zn^2+^ co-intercalation/deintercalation, dissolution–precipitation, and conversion reaction mechanisms [15,16,17]. The performance of cathode materials is closely related to their crystal structures [12]. α-MnO_2_ has become the most widely studied manganese-based cathode material due to its unique tunnel structure [18], but how to optimize its ion diffusion efficiency and mechanical stability through structural design remains a key challenge [19]. In addition, the mechanical and electrochemical properties of flexible electrodes are highly dependent on structural parameters and preparation processes. Structural parameters such as the number of electrode layers, line spacing, and current collector thickness directly affect stress distribution and ion transport efficiency, while binder selection, slurry ratio, and printing parameters determine the forming quality and structural stability of electrodes [10].

Based on the above research background, this study focuses on the preparation and performance regulation of 3D-printed MnO_2_/acetylene black flexible cathodes. Furthermore, the mechanical and electrochemical properties of electrodes with optimal structures were investigated and compared with traditional coated electrodes. Meanwhile, manganese-based aqueous zinc-ion flexible batteries were assembled to verify their power supply stability under different deformation states.

## 2. Materials and Methods

### 2.1. Fabrication of 3D-Printed Zn-Mn Flexible Batteries

#### 2.1.1. Positive Electrode Material Preparation

The main materials used in the flexible Zn-Mn battery cathode include: MnO_2_ powder (≥92.2%, Kelude New Energy Technology Co., Ltd., Dongguan, China) as the cathode active material, acetylene black (≥99.9%, Kappa 100, Kelude New Energy Technology Co., Ltd., Dongguan, China) as the conductive agent, PVDF (model 5130, Aweison Chemical Technology Co., Ltd., Tianjin, China) as the binder, and stainless steel foil (≥99.99%, Kelude New Energy Technology Co., Ltd., Dongguan, China) as the current collector. PVDF needs to be dissolved in NMP (Aweison Chemical Technology Co., Ltd., Tianjin, China) solvent to form a PVDF solution, and the concentration of PVDF solution is 100 mg/mL. The optimal ratio of the electrode slurry is MnO_2_: acetylene black: PVDF = 7:2:1 (mass ratio). The optimization process of the slurry composition, including the effects of different PVDF concentrations on electrode printability and structural stability, is detailed in Supplementary Material (Figure S3).

**Figure 1:**
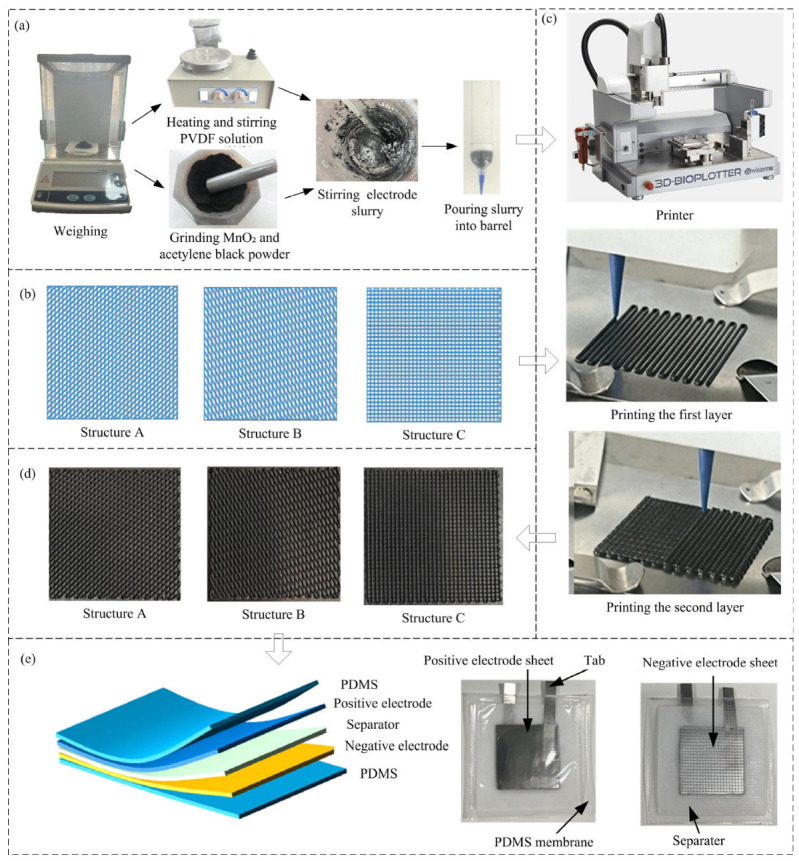
Fabrication of 3D-printed Zn-Mn flexible batteries: (**a**) positive electrode material preparation, (**b**) positive electrode structure design, (**c**) 3D printing process of positive electrodes, (**d**) positive electrode sheet printed samples (**e**) structure and assembled samples of Zn-Mn batteries.

Weigh 2.8 g of MnO_2_ powder and 0.8 g of acetylene black powder with an electronic balance, and grind them in an agate mortar for 40 min. Weigh 0.4 g of PVDF powder, measure 4 mL of NMP liquid into a beaker, mix them, and heat and stir on a magnetic stirrer until the PVDF powder is completely dissolved to form a transparent solution. Finally, mix the ground powder with the transparent solution and stir thoroughly until uniform. Load the prepared electrode slurry into a barrel for electrode printing, as shown in Figure 1a. Notably, PVDF grade 5130 was selected as the optimal binder after comparing its performance with PVDF HSV900, and the detailed comparison experiment and results are provided in Supplementary Material (Figure S4).

#### 2.1.2. Positive Electrode Structure

Both the length and width of the positive electrode are 35 mm. The positive electrode was designed with 2 layers, and a current collector thickness of 0.1 mm. The angles between lines of adjacent electrode layers were 45°, 30°, and 90°, which were defined as structures A (45°), B (30°), and C (90°), respectively, and the line spacing with each layer was 1 mm, as shown in Figure 1b.

#### 2.1.3. Positive Electrodes 3D Printing

A 3D-Bioplotter printer (EnvisionTEC, Gladbeck, Germany) was used for positive electrode printing with a needle diameter of 400 μm. The size of the printed model was 35 mm × 35 mm × 0.64 mm. The model was established using Solidworks and saved in STL format, sliced with Perfactory Start Center software (Version 3.2.1531, EnvisionTEC GmbH, Gladbeck, Germany) with a slice height of 320 μm, imported into VisualMachines software (Version 1.0.25, EnvisionTEC GmbH, Gladbeck, Germany) for parameter setting.

Fix the current collector (stainless steel foil) on the printing platform, with a size of 50 mm × 50 mm. Set the printing parameters as pressure of 4.5 bar, print head moving speed of 7 mm/s, extrusion height of 0.30 mm, and start printing, as shown in Figure 1c. After the electrode printing is completed, place it in a vacuum drying oven and dry at 35 °C for 16 h. Cut the dried electrode sheet into a size of 35 mm × 35 mm, as shown in Figure 1d.

#### 2.1.4. Battery Assembly and Encapsulation

Using 3D-printed electrodes (three structures, A, B, and C) and the coated electrode as the positive electrode separately and zinc foils as the negative electrode, four flexible batteries were assembled. Cut the zinc foil into square sheets of 35 mm × 35 mm, wipe the surface of the electrode sheet with absolute ethanol, and dry it for later use. Using glass fiber (GF/D, Ouleji New Materials Co., Ltd., Chongqing, China) as the separator, 2 M ZnSO_4_ + 0.2 M MnSO_4_ (AR, Sinopharm Chemical Reagent Co., Ltd., Shanghai, China) mixed solution as the electrolyte, and PDMS film (Visichuang Technology Co., Ltd., Hangzhou, Zhejiang, China) as the outer packaging, manganese-based aqueous zinc-ion pouch batteries were assembled (Figure 1e). The list of battery assembly materials is shown in Table 1.

The battery encapsulation steps were as follows: (1) weld the tabs (Ni, Topu Electronics Co., Ltd., Dongguan, Guangdong, China) to the positive and negative electrode sheets, keeping the distance between the white glue edge of the tab and the edge of the electrode sheet at 1 cm; (2) cut the separator into a square of 55 mm × 55 mm (10 mm wider than the electrode sheet on each side), and cut the PDMS film into a square of 75 mm × 75 mm; (3) stack and assemble in the order of PDMS film, cathode sheet, separator, anode sheet, and PDMS film, ensuring that the four sides of the positive and negative electrode sheets are aligned; (4) use a vacuum sealer to heat-seal three sides of the battery first, with a sealing width of about 3 mm, leaving the tab end for electrolyte injection; (5) inject an appropriate amount of electrolyte into the battery from the tab end. After the separator is completely wetted, encapsulate the fourth side and let it stand for more than 8 h for later use.

### 2.2. Performance Test

#### 2.2.1. Mechanical Experiment

**Figure 2:**
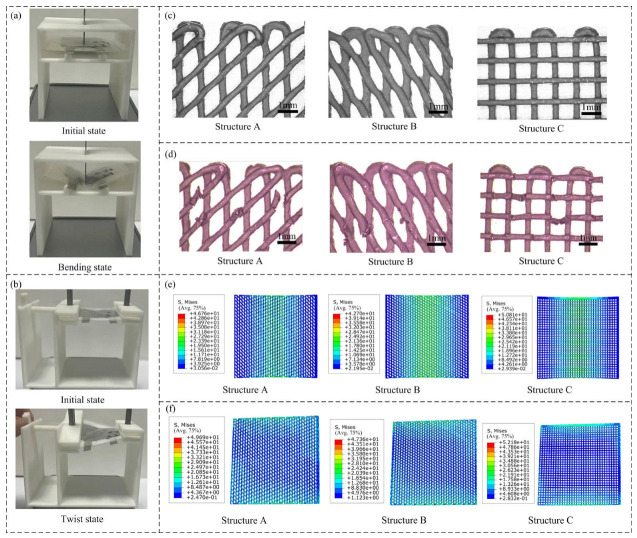
Mechanical experiment and results: (**a**) bending experiment, (**b**) twist experiment, (**c**) partially magnified micrograph of positive electrode before experiment, (**d**) partially magnified micrograph of positive electrode after experiment, (**e**) FEM stress analysis results of bending experiment, (**f**) FEM stress analysis results of twist experiment.

A bending and torsion deformation test bench was designed to conduct mechanical performance tests on batteries assembled with three types of structure electrodes A (angle 45°), B (angle 30°), and C (angle 90°). In the bending test, both sides of the battery were fixed, and the central part was pressed downward with a displacement of 5 mm to induce bending (Figure 2a), which was repeated 200 times. In the torsion test, one end of the battery was fixed, and the other end was rotated to 30° (Figure 2b), with the process repeated 200 times. The morphologies of the electrode before and after deformation were observed using an electron microscope (ZOOM-2860, Wumo Optical Instrument Co., Ltd., Shanghai, China), as shown in Figure 2c,d. Detailed morphological images of the three electrode structures before and after mechanical tests, including the upper, middle, and lower sections, are provided in Supplementary Figures S1 and S2, which clearly show the structural damage differences among the three samples.

Finite element simulations of bending and torsion under the same experimental procedures and conditions as described above were also conducted to analyze the mechanical performance of the three 3D-printed battery structures; see Figure 2e,f.

#### 2.2.2. Electrochemical Test

**Figure 3:**
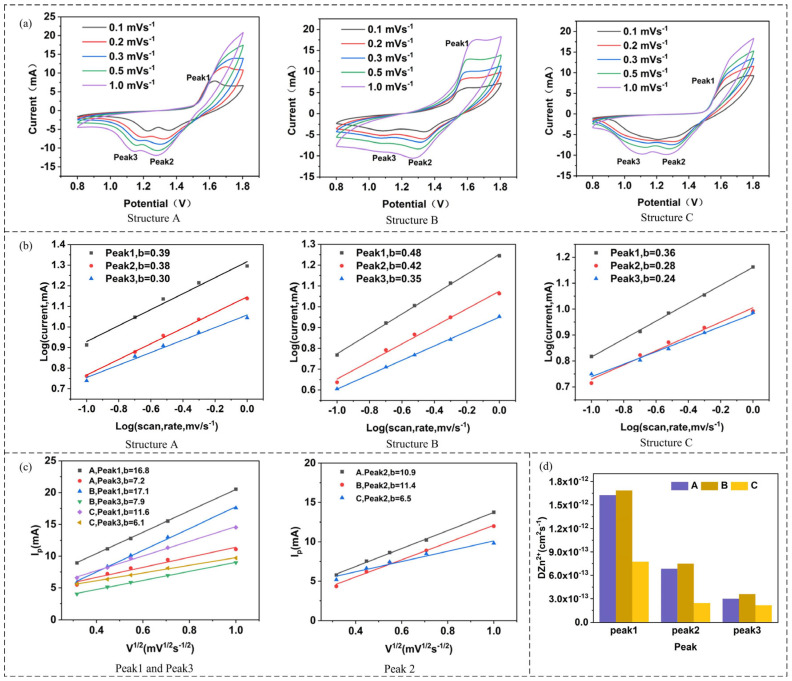
Cyclic voltammetry test results of flexible batteries: (**a**) CV curves at different sweep speeds, (**b**) relationship between Log (*i*) and Log (*v*), (**c**) relationship between *I*_p_ and *v*^1/2^, (**d**) diffusion coefficients at different redox peaks.

**Figure 4:**
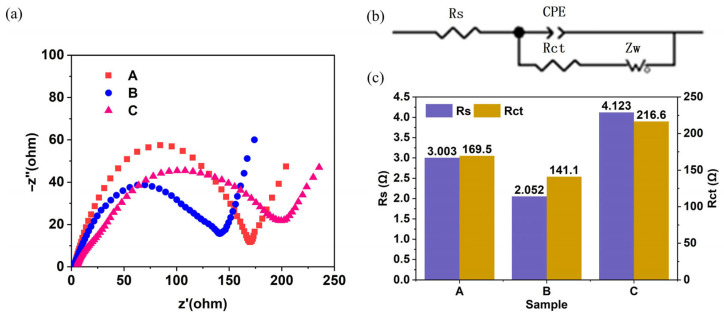
Electrochemical impedance spectroscopy test results. (**a**) Electrochemical impedance spectrum of 3D-printed electrode, (**b**) 3D-printed electrode equivalent circuit diagram, (**c**) fitting results of 3D-printed electrode equivalent circuit.

**Figure 5:**
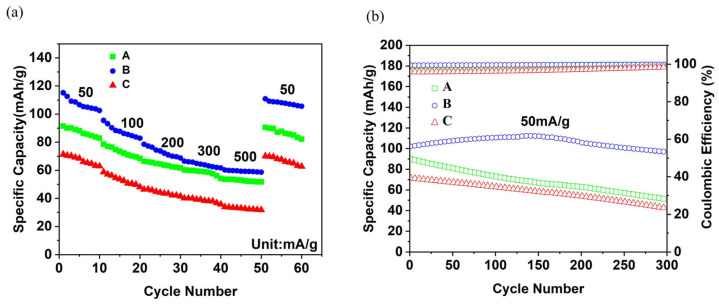
Galvanostatic charge–discharge test results. (**a**) Cyclic performance of electrodes of A, B and C structures at different current densities, (**b**) cyclic performance of A, B and C electrodes at 50 mA/g current density.

To investigate the electrochemical reaction kinetics and the control mechanism of electrode reactions for different electrode structures, analyze the effect of electrode structure on electrochemical impedance, and evaluate the performance of electrodes in terms of capacity and cycling stability, cyclic voltammetry (CV) tests (Figure 3), electrochemical impedance spectroscopy (EIS) tests (Figure 4), and galvanostatic charge–discharge tests (Figure 5) were performed separately on the 3D-printed electrodes with structures A, B, and C. The EIS test results of the three electrodes after mechanical deformation, including the Nyquist plots and equivalent circuit fitting data, are supplemented in Supplementary Material (Figure S5 and Table S1), which further verifies the effect of electrode structure on ionic transport and charge transfer efficiency after deformation.

A CS350M electrochemical workstation was used for CV testing (voltage window 0.8–1.8 V, scan rate 0.1–1.0 mV/s) and EIS testing (frequency range 0.01–100,000 Hz, amplitude 5 mV); a CT3002A battery test system was used for galvanostatic charge–discharge testing (voltage window 0.8–1.8 V, current density 50–500 mA/g).

#### 2.2.3. Performance Comparison

**Figure 6:**
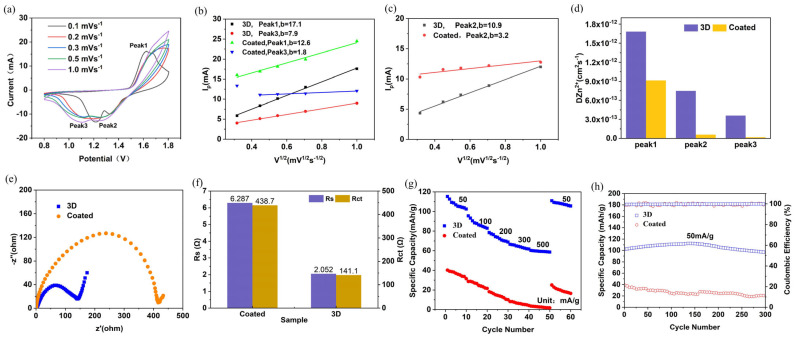
3D-printed and coated electrodes comparison results. (**a**) CV curves of coated electrodes at different sweep speeds, (**b**) relationship between *I_p_* and *v*^1/2^ at Peak 1 and Peak 3, (**c**) relationship between *I_p_* and *v*^1/2^ at Peak 2, (**d**) diffusion coefficients under different REDOX peaks, (**e**) electrochemical impedance spectra, (**f**) fitting results of equivalent circuit between 3D-printed electrode and coated electrode, (**g**) cyclic performance at different current densities, (**h**) cycling performance at 50 mA/g current density.

To investigate whether 3D-printed electrodes exhibit superior electrochemical performance to electrodes fabricated by the coating method, electrode sheets with the same dimensions as the 3D-printed electrodes were prepared via the coating process. Cyclic voltammetry, electrochemical impedance spectroscopy, and galvanostatic charge–discharge tests were conducted, and the results were compared with those of the 3D-printed electrodes (Figure 6).

### 2.3. Power Supply Experiment

**Figure 7:**
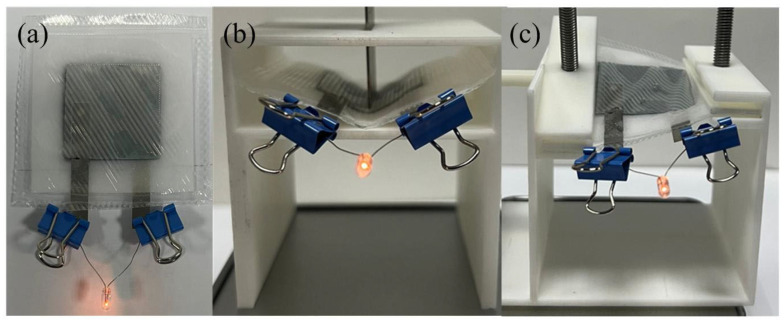
Power supply of flexible batteries: (**a**) flat state, (**b**) bent state, (**c**) twisted state.

To test the potential for practical application of 3D-printed flexible electrodes, the tabs of the flexible battery pouch assembled with the B-structure anode were connected to a 1.5 V LED bulb (1.5V, Xingshen Lamp Factory, Shanghai, China) for a power supply experiment (Figure 7).

## 3. Results

### 3.1. Mechanical Experiment Results

As shown in Figure 2d, the structure A and C electrodes showed obvious fracture and damage after experiment, while the structure B electrode only had slight creases and the minimum morphological change; the simulation results of the bending and torsion tests indicated that electrode B exhibited the minimum stress value (Figure 2e,f), which was consistent with the micromorphology observations (Figure 2d). Supplementary Figures S1 and S2 provide more detailed morphological evidence. Figure S1 shows the intact grid structure of the three electrodes before mechanical tests, confirming their good initial printability; Figure S2 clearly displays the structural damage of the three electrodes after 200 cycles of bending–torsion tests, further verifying that the B-structured electrode has the best mechanical stability.

### 3.2. Electrochemical Test Results

#### 3.2.1. Cyclic Voltammetry Test

Figure 3a shows the CV curves of the 3D-printed electrodes with structures A, B, and C at scan rates of 0.1 mV/s, 0.2 mV/s, 0.3 mV/s, 0.5 mV/s, and 1.0 mV/s. Three distinct peaks appear in each CV curve. The initial reduction peak, Peak 2, corresponds to the intercalation of H^+^, and the initial reduction peak, Peak 3, corresponds to the intercalation of Zn^2+^; the initial oxidation peak, Peak 1, corresponds to the deintercalation of Zn^2+^.

To investigate the energy storage mechanism of the three 3D-printed electrodes, the peak current (*i_p_*) and scan rate (*v*) were fitted according to Equation (1):

*i_p_* = *av^b^*, (1)

where *v* and *i_p_* are the scan rate and the corresponding peak current, respectively, and *a* and *b* are constants. To obtain the specific value of *b*, the logarithmic transformation of Equation (1) was performed to yield Equation (2):

*logi_p_* = *blogv* + *loga*, (2)

Based on Equation (2), logarithmic linear fitting was performed on the peak current data at different scan rates obtained from Figure 3a. The *b*-values corresponding to the oxidation and reduction peaks of the 3D-printed electrodes with structures A, B, and C are shown in Figure 3b. According to the fitted *b*-values (greater than 0.5), the energy storage mechanisms of all three 3D-printed electrodes (A, B, and C) are dominated by diffusion control.

In 3D-printed electrodes, the electrode structure exerts a significant influence on the ion diffusion coefficient. Therefore, to further analyze the ion diffusion coefficients of electrodes A, B, and C, linear fitting was conducted between the peak current *i_p_* and the square root of the scan rate *v*^1/2^ based on Equation (3), with the results presented in Figure 3c:

*i_p_* = 2.69 × 10^5^ × *A* × *n*^3/2^ × Δ*C* × *D*^1/2^ × *v*^1/2^, (3)

where *i_p_* is the peak current (mA), *A* is the electrode area (cm^2^), *A* = 12.25 cm^2^; *n* is the number of transferred electrons in the redox reaction, *n* = 2; *C* is the concentration of the reactant in the electrolyte (mol/cm^3^), *C* = 2 mol/cm^3^; *D* is the ion diffusion coefficient (cm^2^/s).

It can be observed from Figure 3c that the slope of the fitted line follows the order B > A > C, indicating that ion diffusion is more favorable in the B-structured electrode. To more intuitively compare the ion diffusion coefficients of the three electrodes at different redox peaks, the ion diffusion coefficients D were calculated using Equation (3), as displayed in Figure 3d. It clearly shows that the Zn^2+^ diffusion coefficient of the B-structured electrode is the largest, demonstrating that the B-structured battery possesses the lowest ion transport resistance and the best kinetic performance.

#### 3.2.2. Electrochemical Impedance Spectroscopy Test

Figure 4a shows the Nyquist plots of the electrochemical impedance spectra for the electrodes with structures A, B, and C. The abscissa *Z*′ represents the real part of the electrochemical impedance, and the ordinate *Z*″ represents the imaginary part. The Nyquist curves mainly consist of a semicircle in the medium-frequency region and a straight line in the low-frequency region. The measurements were conducted at a frequency range of 0.01–100,000 Hz with an amplitude of 5 mV.

The equivalent circuit model of the electrode was fitted based on the shape of the Nyquist plots, and the structure of the equivalent circuit is displayed in Figure 4b. Here, *R_s_* is the ohmic resistance (in the ultrahigh-frequency region), representing the resistance in the absence of charge transfer; *R_ct_* is the interfacial charge transfer resistance (in the medium-frequency region, corresponding to the semicircle), representing the resistance of charge transfer from the electrode to the electrolyte during the electrochemical reaction; CPE is the constant phase element; and *Z_w_* is the Warburg impedance (in the low-frequency region, corresponding to the straight line), representing the impedance caused by ion diffusion in the electrolyte, i.e., the ion diffusion resistance.

Figure 4c presents the specific values of *R_s_* and *R_ct_* for the electrodes with different structures obtained by fitting the equivalent circuit. It can be seen that both the ohmic resistance and the interfacial charge transfer resistance of the B-structured electrode are lower than those of the A-structured and C-structured electrodes, indicating that the conductive network formed by the B-structured electrode exhibits higher conductivity and promotes the ion transport process at the electrode/electrolyte interface. The Supplementary Materials (Figure S5 and Table S1) provide the EIS test results of the three electrodes after mechanical deformation, showing that the B-structured electrode still maintains lower Rs and Rct increase rates after deformation, further confirming its excellent structural stability and ion transport performance.

#### 3.2.3. Galvanostatic Charge–Discharge Test

Figure 5a shows that the specific capacities of the electrodes with structures A, B, and C are 92 mAh/g, 114 mAh/g, and 71 mAh/g at a current density of 50 mA/g, and 51 mAh/g, 65 mAh/g, and 29 mAh/g at 500 mA/g, respectively. It can be observed that the capacity retention of the B-structured electrode is higher than those of the A- and C-structured electrodes with increasing current density.

Figure 5b shows that the initial specific capacities of the A, B, and C electrodes are 91 mAh/g, 103 mAh/g, and 72 mAh/g, and the specific capacities after 300 cycles are 55 mAh/g, 95 mAh/g, and 42 mAh/g, respectively. The capacity retentions of the A, B, and C electrodes at 50 mA/g are calculated to be 60.4%, 92.2%, and 58.3%, respectively. It is clear that the long-cycle performance of the B-structured electrode is superior to those of the A- and C-structured electrodes.

These results demonstrate that the B-structured electrode possesses higher specific capacity and better cycling stability. The excellent performance of the B-structured electrode is closely related to its optimal structural design and the rational selection of slurry components, which is further supported by the supplementary experimental data in the Supplementary Materials (Figures S3 and S4).

### 3.3. Comparison of 3D-Printed and Coated Electrodes

Figure 6a shows the CV curves of coated electrodes at different sweep speeds. Figure 6b–d shows the comparative results of cyclic voltammetry analysis for 3D-printed and coated batteries. It can be seen that the diffusion coefficient of the 3D-printed electrode is much higher than that of the coated electrode.

Figure 6e displays the Nyquist plots of the 3D-printed and coated electrodes. The two curves exhibit similar shapes, both consisting of a semicircle in the medium-frequency region and an inclined line in the low-frequency region. Figure 6f presents the ohmic resistance *R_s_* and interfacial charge transfer resistance *R_ct_* of the 3D-printed and coated electrodes. It is clear that both the *R_s_* and *R_ct_* of the coated electrode are significantly larger than those of the 3D-printed electrode, indicating that the 3D-printed electrode possesses higher electrical conductivity.

Figure 6g depicts the rate cycling performance of 3D-printed and coated electrodes at various current densities, and Figure 6h presents the cycling curves of 3D-printed and coated electrodes at a current density of 50 mA/g for 300 cycles. It can be observed that the specific capacity of the 3D-printed electrode is much higher than that of the coated electrode at both low and high current densities. Moreover, the capacity retention of the 3D-printed electrode is superior to that of the coated electrode with increasing current density and long-term charge–discharge cycling.

This phenomenon can be attributed to the higher specific surface area of the 3D-printed electrode, which facilitates faster ion diffusion and enhanced charge transport efficiency. The advantages of 3D-printed electrodes become particularly prominent at high rates, where the demand for ion and electron transport is significantly increased. In 3D-printed electrodes, the electrolyte can penetrate and distribute more uniformly into the interior of the electrode, reducing the resistance during charge transfer and enabling faster reaction kinetics.

In contrast, conventional coated electrodes usually possess a two-dimensional or relatively simple surface structure, which cannot provide sufficient electrolyte contact sites and charge transfer pathways. This results in slower ion diffusion within the coating layer. Under high-rate conditions, ions cannot rapidly reach the deep region of the electrode, leading to increased internal resistance and limited rate capability of the coated electrode.

### 3.4. Power Supply Experiment Results

As can be seen from Figure 7, the LED bulb lights up successfully whenever the battery is in the flat, bent or twisted states. Moreover, it can still supply power normally after repeated bending and twisting for 200 cycles, indicating that the prepared flexible electrode exhibits excellent mechanical properties. This result is consistent with the morphological observation results in Supplementary Figures S1 and S2, confirming that the B-structured electrode can maintain structural integrity under mechanical deformation, thus ensuring stable power supply performance.

## 4. Discussion

In this study, high-performance 3D-printed MnO_2_/acetylene black flexible cathodes were successfully prepared. The optimal structure electrode (two layers, line spacing of 1 mm, current collector thickness of 0.1 mm, and angle of 30° between lines of adjacent layers) achieved synergistic improvement in mechanical and electrochemical properties. The reasons can be concluded that the reasonable number of layers and line spacing balanced the mechanical strength and flexibility of the electrode, the 30° angle design reduced stress concentration, and the 0.1 mm thick current collector reduced the electrode rigidity while ensuring conductivity. In terms of process, the selection of PVDF (model 5130) solved the problem of electrode fracture during drying, the concentration of 100 mg/mL PVDF solution ensured the rheological properties and printability of the slurry, the combination of 4.5 bar pressure, 7 mm/s moving speed, and 0.30 mm extrusion height achieved uniform and continuous line printing, and the drying temperature of 35 °C avoided electrode cracking and solvent residue.

Compared with traditional coated electrodes, the advantages of 3D-printed electrodes are mainly reflected in three aspects. First, the porous structure increases the specific surface area and electrolyte contact area, improving the ion diffusion coefficient; second, the three-dimensional network structure improves the electronic conduction path, reducing the ohmic resistance and interfacial charge transfer resistance; third, the combination between the electrode and the current collector is tighter, with stronger mechanical stability and less structural damage during cycling. These factors together result in the 3D-printed electrode being significantly superior to the coated electrode in specific capacity, rate performance, and cycle stability.

The power supply experiment of the flexible battery showed that the battery assembled with the optimal structure electrode could stably power the LED bulb under flat, bent, and twisted states, verifying its application potential in flexible electronic devices. However, this study still has certain limitations: the number of bending and twisting experiments (200 times) is relatively less than the requirements of practical applications. In the future, a dedicated test bench can be used to increase the number of cycles to verify long-term stability; the electrochemical performance of the electrode is still inferior to that of lithium-ion batteries. In the future, the performance can be further improved by optimizing the electrode material composite method and introducing ion-conductive additives.

## 5. Conclusions

To address the energy storage demands of flexible wearable electronic devices, this study systematically optimized the preparation and performance of 3D-printed MnO_2_/acetylene black flexible cathodes and successfully assembled manganese-based aqueous zinc-ion flexible batteries, yielding key findings that link process, structure, performance, and application.

PVDF (model 5130) was confirmed as the optimal binder for cathode fabrication, with the electrode slurry formulated at a mass ratio of MnO_2_: acetylene black: PVDF = 7:2:1 (PVDF concentration: 100 mg/mL). For 3D printing, parameters of 4.5 bar pressure, 7 mm/s moving speed, and 0.30 mm extrusion height were identified to balance extrusion uniformity and structural integrity; vacuum drying at 35 °C further ensured the best forming quality, eliminating defects such as line breakage or uneven extrusion that could compromise performance.

Among the tested structural configurations, the electrode with two layers, 1 mm line spacing, 0.1 mm current collector thickness, and a 30° angle between lines of adjacent layers stood out for mechanical stability. After 200 cycles of bending and twisting tests, this electrode showed the minimum structural damage, maintaining its integrity better than counterparts with different layer counts, line spacings, or interlayer angles.

The cathode with the optimized process and structure also exhibited excellent electrochemical performance: it had an ohmic resistance of 2.052 Ω and an interfacial charge transfer resistance of 141.1 Ω, facilitating efficient ion and electron transport. Galvanostatic charge–discharge tests revealed a specific capacity of 103 mAh/g at 50 mA/g and 65 mAh/g at 500 mA/g, with all these metrics outperforming both non-optimally structured electrodes and traditional coated electrodes.

When integrated into a manganese-based aqueous zinc-ion flexible battery, the optimized cathode enabled stable power supply to a 1.5 V LED bulb under flat, bent, and twisted states, confirming its adaptability to the deformation requirements of wearable devices. This work not only verifies the application potential of 3D-printed electrodes in flexible electronics but also provides a feasible technical scheme for advancing the development of high-performance flexible energy storage devices.

Notably, the adaptability of the 3D-printed electrode in this study to low-power wearable scenarios is further reflected in the synergy of “performance–cost–scalability”. Compared with the 3D conductive interface modification relied on in the literature [20], which requires multi-step hydrothermal and coating processes and incurs high costs, and the complex 3D structure forming method in the literature [21], the fused deposition modeling (FDM) 3D printing technology adopted in this study can directly shape the MnO_2_/acetylene black composite slurry into the target structure. This reduces the single-electrode preparation time to 15 min and achieves a material utilization rate of over 98%, significantly lowering the threshold for large-scale applications. Although the ultrahigh specific capacity of Literature [1] and the high current tolerance of Literature [2] are more suitable for high-power scenarios, the “high loading–flexibility–low cost” balance achieved by this study through structural design provides a more practical technical route for the transition of MnO_2_-based flexible ZIBs to wearable mass production applications.

## Figures and Tables

**Table 1:** Battery assembly materials.

Material	Molecular Formula or Abbreviation	Specification	Manufacturer
Manganese dioxide	MnO_2_	≥92.2%	Kelude, Dongguan, GD, China
Acetylene black (Kappa 100)	C	≥99.9%	Kelude, Dongguan, GD, China
Stainless steel foil	-	≥99.99%	Kelude, Dongguan, GD, China
Polyvinylidene fluoride	PVDF	HSV900/5130	Aweison, Tianjin, China
N-methylpyrrolidone	NMP	-	Aweison, Tianjin, China
Zinc foil	Zn	≥99.99%	Aweison, Tianjin, China
Glass fiber separator	-	GF/D	Ouleji, Chongqing, China
Nickel tab	Ni	Width 5 mm	Topu Electronics, Dongguan, GD, China
Manganese sulfate monohydrate	MnSO_4_·H_2_O	AR	Sinopharm, Shanghai, China
Zinc sulfate heptahydrate	ZnSO_4_·7H_2_O	AR	Sinopharm, Shanghai, China
Deionized water	H_2_O	Ultrapure	Sinopharm, Shanghai, China
Polydimethylsiloxane	PDMS	-	Visichuang, Hangzhou, ZJ, China
LED bulb	-	1.5 V	Xingshen, Shanghai, China
Absolute ethanol	CH_3_CH_2_OH	≥99.5%	Aladdin Reagent, Shanghai, China

## Data Availability

The original contributions presented in this study are included in the article/Supplementary Material. Further inquiries can be directed to the corresponding author.

## Supplementary Materials

The following supporting information can be downloaded at https://www.mdpi.com/article/10.3390/ma19071309/s1, Figure S1: Micro-morphology of 3D-printed electrodes before mechanical tests; Figure S2: Micro-morphology of 3D-printed electrodes after 200 cycles of bending and torsion tests; Figure S3: Structural morphology of 3D-printed electrodes before and after drying: (a) electrodes printed from Slurry A (100 mg/mL PVDF), (b) electrodes printed from Slurry B (80 mg/mL PVDF, yellow boxes indicate cracking), (c) electrodes printed from Slurry C (60 mg/mL PVDF, red boxes indicate delamination and curling); Figure S4: Morphological images of 3D-printed electrodes after drying with different PVDF grades: (a) Electrode printed with PVDF HSV900; (b) Electrode printed with PVDF 5130; Figure S5: Electrochemical impedance diagram (Nyquist plots) of 3D-printed electrodes after deformation; Table S1: Equivalent circuit fitting results after deformation of 3D printed electrode.
