# Supplementary material for: Fabrication and Performance Evaluation of 3D-Printed Zinc–Manganese Flexible Batteries
Source: Materials (Basel). 2026 Mar 26;19(7):1309. doi: 10.3390/ma19071309 (PMC13073813; doi:10.3390/ma19071309)
Supplement: Supplementary file 1 [file materials-19-01309-s001.zip › materials-4209813-supplementary.pdf]

Fabrication and Performance Evaluation of 3D-Printed Zinc-Manganese Flexible Batteries

Ernan Ju[1,2], Cong Yan[2], Li Wu[2,*]

[1] Dalian Neusoft University of Information, Department of Electronic Engineering, Dalian, Liaoning, China; juernan@neusoft.edu.cn
[2] Dalian Jiaotong University, Institute of Mechanical Engineering, Dalian, Liaoning, China;
[*] Correspondence: wuli@djtu.edu.cn; djtuwuli@163.com

1. Morphology of the electrode before and after mechanical property test

Figure S1 displays the optical micrographs of the 3D-printed grid electrodes (Samples A, B, and C) before mechanical tests, focusing on their initial structural integrity. All three samples exhibit well-formed grid structures with uniform line widths and well-distributed pores, without any cracks, delamination, or structural defects. This confirms that the optimized 3D printing parameters and slurry formulation (MnO_2 : acetylene black : PVDF = 7:2:1, with NMP as the solvent) ensure good printability and initial structural stability, laying a foundation for subsequent mechanical and electrochemical performance tests.

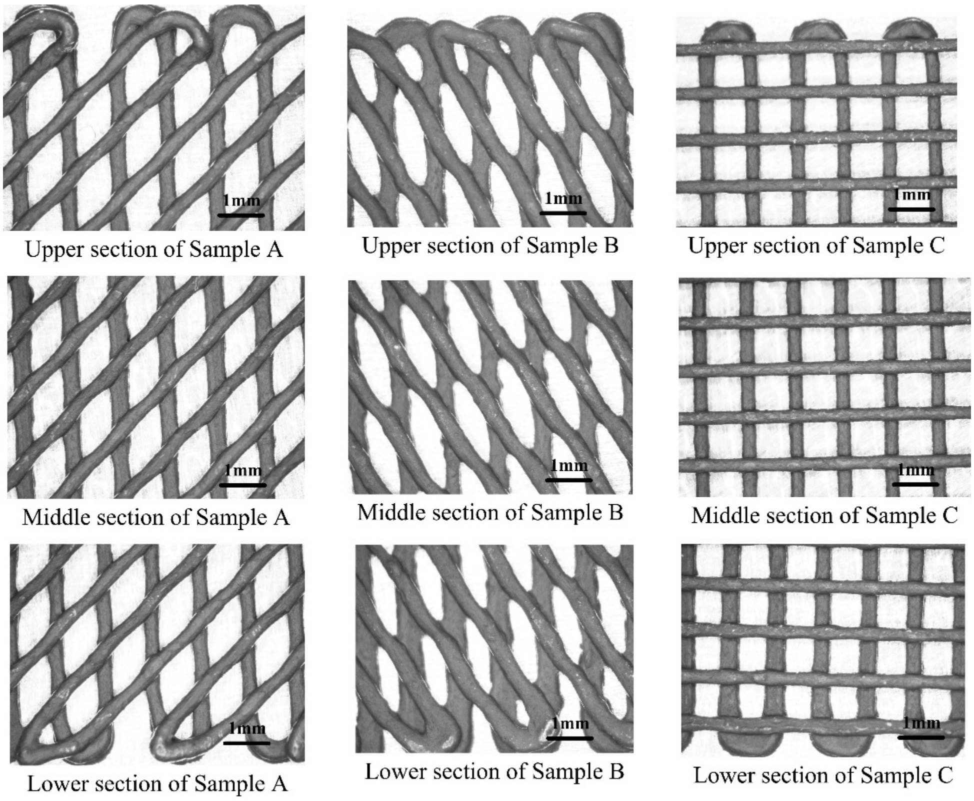

Figure S1. Micro-morphology of 3D-printed electrodes before mechanical tests

Figure S2 presents the optical micrographs of the 3D-printed electrodes after 200 cycles of bending-torsion tests, aiming to visually reflect the structural stability differences among the three samples. For Sample A and Sample C, line shrinkage and local cracking appear at the grid joints; for Sample B, the grid structure remains intact with slight damage—little line

breakage, delamination, or pore collapse is observed, indicating excellent mechanical flexibility and structural reliability.

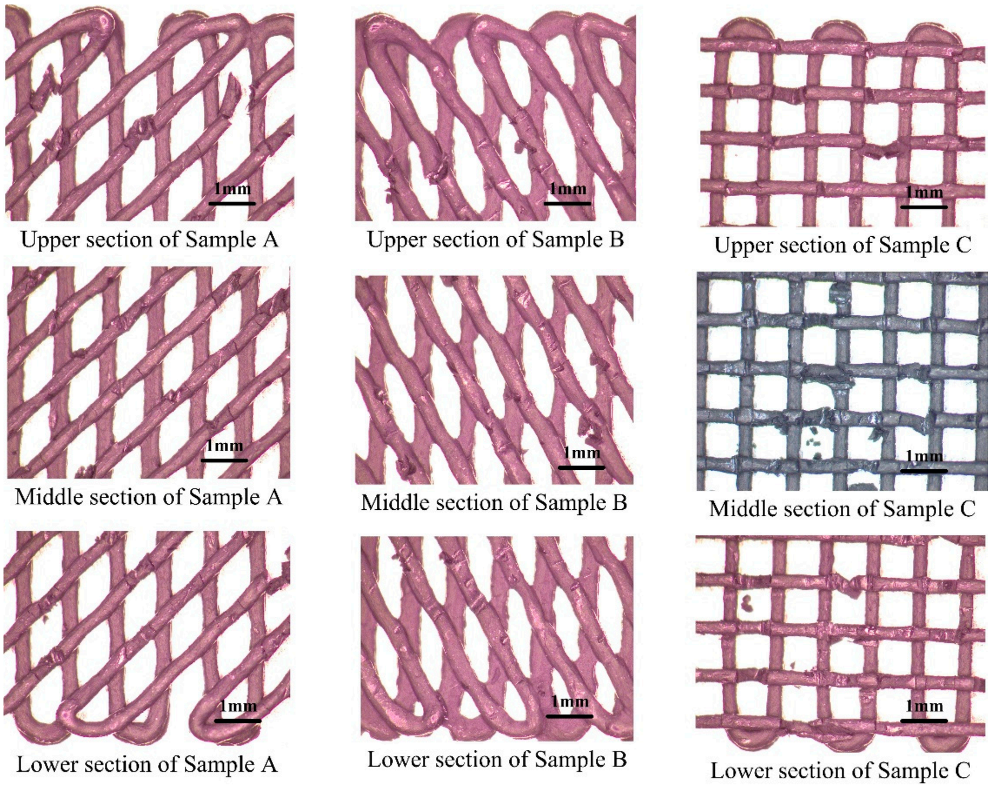

Figure S2. Micro-morphology of 3D-printed electrodes after 200 cycles of bending and torsion tests.

2. Optimization of Electrode Slurry Composition (MnO_2 : Acetylene Black : PVDF Ratio)

2.1 Experimental Procedure for Slurry Preparation

To determine the optimal electrode slurry composition for 3D printing, three formulations with varying PVDF content were prepared, following the steps below:

(1) Base powder mixture: 2.8 g commercial α-MnO_2 powder (purity ≥ 99%) and 0.8 g acetylene black powder were mixed and ground in an agate mortar for 40 min to ensure uniform dispersion.

(2) PVDF binder solutions: Three PVDF/NMP solutions were prepared by dissolving 0.40 g, 0.32 g, and 0.24 g PVDF powder (grade 5130) in 4 mL N-methylpyrrolidone (NMP, analytical purity) under magnetic stirring at 60 °C, yielding concentrations of 100 mg/mL, 80 mg/mL, and 60 mg/mL, respectively.

(3) Slurry formulation: The ground MnO_2/acetylene black mixture was added to each PVDF solution and stirred for 2 h to form homogeneous electrode slurries, denoted as Slurry A (100 mg/mL PVDF), Slurry B (80 mg/mL PVDF), and Slurry C (60 mg/mL PVDF). The corresponding mass ratios of MnO_2 : acetylene black : PVDF were 7:2:1 (A), 7:2:0.8 (B), and 7:2:0.6 (C).

2.2 3D Printing and Drying Process

Electrode sheets with 2, 3, and 4 layers were printed from each slurry using fused deposition modeling (FDM) 3D printing, with consistent parameters: nozzle diameter 0.4 mm,

nozzle temperature 25 °C, printing speed 5 mm/s, layer height 0.1 mm. All printed electrodes were vacuum-dried at 80 °C for 12 h to remove residual NMP solvent. The structural integrity of each electrode was observed before and after drying, and the results are summarized in Figure S3.

2.3 Structural Stability Evaluation

Before drying: All electrodes printed from Slurries A, B, and C maintained intact 3D lattice structures without collapse or deformation, demonstrating good printability for all formulations.

After drying: Shrinkage and thinning of printed lines were observed in all samples due to solvent evaporation.

Slurry A: All 2–4 layer electrodes retained complete structural integrity after drying, with no cracking, delamination, or curling observed.

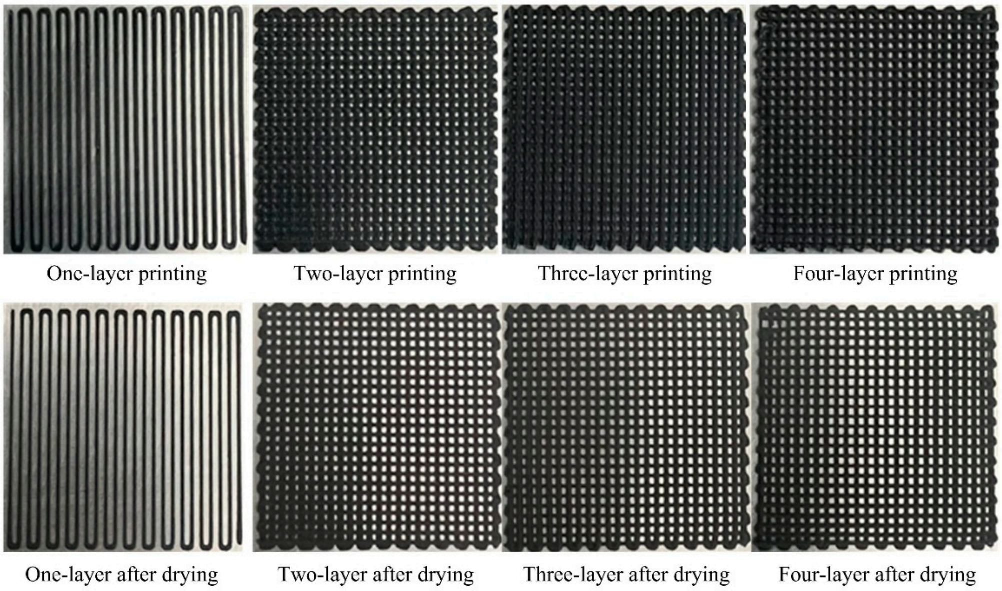

(a)

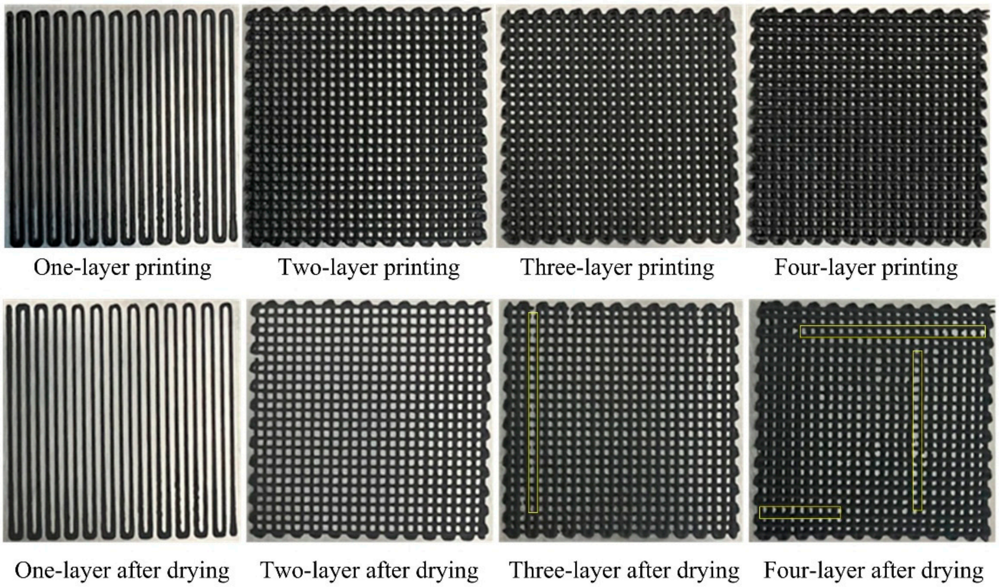

(b)

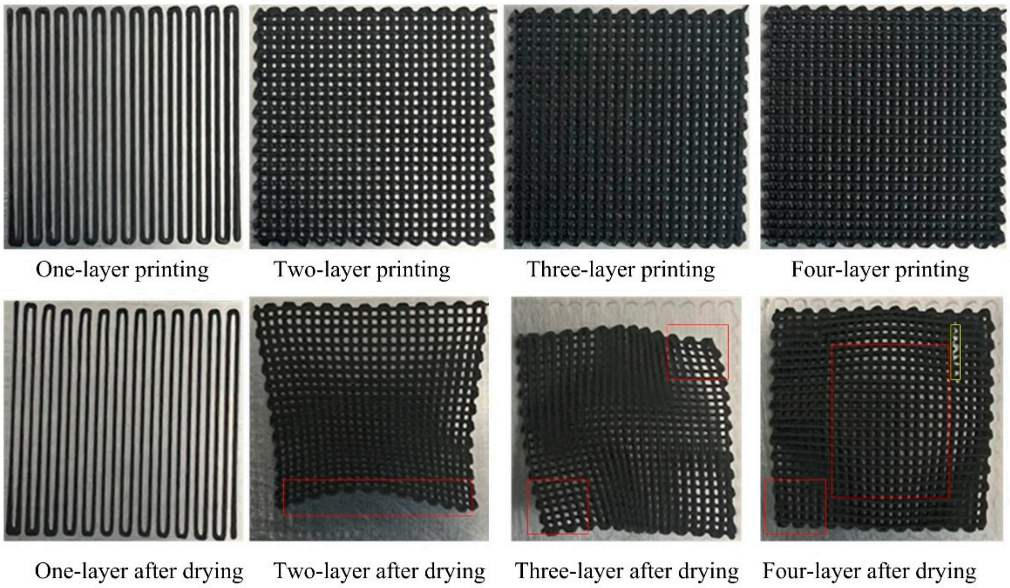

(c)

Figure S3. Structural morphology of 3D-printed electrodes before and after drying: (a) electrodes printed from Slurry A (100 mg/mL PVDF), (b) electrodes printed from Slurry B (80 mg/mL PVDF, yellow boxes indicate cracking), (c) electrodes printed from Slurry C (60 mg/mL PVDF, red boxes indicate delamination and curling).

Slurry B: 3-layer and 4-layer electrodes exhibited obvious cracking (marked by yellow boxes in Figure S3) after drying, indicating insufficient mechanical strength to resist shrinkage stress.

Slurry C: 2–4 layer electrodes detached from the current collector (stainless steel foil) and curled severely (marked by red boxes in Figure S3), indicating poor adhesion between the electrode material and the current collector.

Based on the structural stability after drying, Slurry A (MnO_2 : acetylene black : PVDF = 7:2:1) was selected as the optimal formulation for subsequent electrode fabrication, as it balanced printability, mechanical strength, and adhesion to the current collector.

3. Effect of PVDF Binder Grade on 3D-Printed Electrode Flexibility and Ionic Transport

3.1 Experimental Purpose

Polyvinylidene Fluoride (PVDF) is a commonly used binder in MnO_2-based zinc-ion battery (ZIB) electrodes, and its grade directly affects the printability, mechanical flexibility, and ionic transport performance of 3D-printed electrodes. This experiment aims to compare the effects of two PVDF grades (HSV900 and 5130) on the structural integrity, flexibility, and ionic transport channels of 3D-printed MnO_2 electrodes, providing a basis for the selection of optimal PVDF binder grade.

3.2 Experimental Materials and Equipment

Materials: PVDF (grades HSV900 and 5130), N-methylpyrrolidone (NMP, analytical purity), commercial α-MnO_2 powder (purity ≥ 99%), acetylene black (conductive agent), current collector (stainless steel foil).

Equipment: Magnetic stirrer (with heating function), agate mortar, 3D printer (nozzle diameter 0.4 mm), vacuum drying oven, optical microscope.

3.3 Experimental Procedure

(1) Preparation of PVDF binder solutions: Two PVDF solutions with a concentration of 100 mg/mL were prepared by dissolving PVDF (HSV900 and 5130 grades, respectively) in NMP solvent. Each solution was heated and stirred at 60 °C for 2 h until the PVDF powder was completely dissolved to form a transparent and homogeneous solution.

(2) Preparation of electrode slurries: The base powder mixture (2.8 g MnO_2 + 0.8 g acetylene black) was ground in an agate mortar for 40 min to ensure uniform dispersion. The ground powder mixture was then added to the two PVDF solutions separately, followed by stirring for 2 h to obtain homogeneous electrode slurries (denoted as Slurry HSV900 and Slurry 5130, respectively), with the mass ratio of MnO_2:acetylene black:PVDF maintained at 7:2:1.

(3) 3D printing of electrodes: Two layer electrodes with the same size (35 mm × 35 mm × 0.64 mm) were printed using the two slurries, with consistent 3D printing parameters (nozzle temperature 25 °C, printing speed 5 mm/s, layer height 0.1 mm).

(4) Drying and characterization: All printed electrodes were vacuum-dried at 80 °C for 12 h to remove residual NMP solvent. The morphological characteristics of the electrodes after drying were observed using an optical microscope, and the structural integrity, flexibility, and ionic transport channel status were evaluated.

3.4 Experimental Results and Analysis

Figure S4 shows the morphological images of the 3D-printed electrodes after drying with the two PVDF grades, and the specific performance differences are analyzed as follows.

PVDF HSV900: The electrodes printed from Slurry HSV900 exhibited severe cracking and fracture after drying (as shown in Figure S4a). The main reason is that HSV900 has poor flexibility and low adhesion; during the solvent evaporation process, the internal stress generated by the shrinkage of the electrode cannot be effectively released, leading to structural damage. The fractured structure destroys the continuous ionic transport channels, which will hinder the diffusion of ions between the electrode and the electrolyte, thereby affecting the electrochemical performance of the electrode.

PVDF 5130: In contrast, the electrodes printed from Slurry 5130 maintained intact and uniform morphologies after drying (as shown in Figure S4b), with no visible cracks, fractures, or deformation. This is because PVDF 5130 has excellent flexibility and adhesion, which can effectively buffer the internal stress generated during solvent evaporation, ensuring the structural integrity of the electrode. The intact 3D structure of the electrode maintains unobstructed ionic transport channels, facilitating the rapid diffusion of ions and laying a foundation for good electrochemical performance.

Under the same experimental conditions (100 mg/mL PVDF concentration, same printing and drying parameters), PVDF 5130 grade exhibits superior performance compared to PVDF HSV900 in terms of supporting 3D-printed electrode flexibility and maintaining ionic transport channels. Therefore, PVDF 5130 was selected as the binder for the 3D-printed MnO_2 electrodes in this study, which ensures the structural integrity of the electrodes and the smoothness of ionic transport, further supporting the excellent electrochemical and mechanical performance of the electrodes.

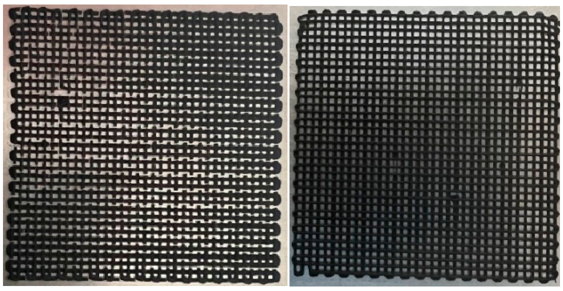

Figure S4. Morphological images of 3D-printed electrodes after drying with different PVDF grades: (a) Electrode printed with PVDF HSV900; (b) Electrode printed with PVDF 5130.

4. Electrochemical Impedance Spectroscopy (EIS) Results After Electrode Deformation

Figure S5 presents the Nyquist plots of the 3D-printed electrodes (Samples A, B, and C) after mechanical deformation, which reflects the ionic transport and charge transfer efficiency of the electrodes. The Nyquist curves consist of a semicircle in the medium-frequency region (corresponding to charge transfer resistance, Rct) and a straight line in the low-frequency region (corresponding to Warburg impedance, Zw, related to ion diffusion). It can be observed that Sample B exhibits the smallest semicircle diameter, indicating the lowest charge transfer resistance and the most efficient ion transport after deformation, while Sample C shows the largest semicircle, suggesting severe structural damage that hinders charge transfer and ion diffusion.

Table S1 summarizes the equivalent circuit fitting results of the Nyquist plots, including the ohmic resistance (R_s), charge transfer resistance (R_{ct}), and their increase rates compared with those before deformation.

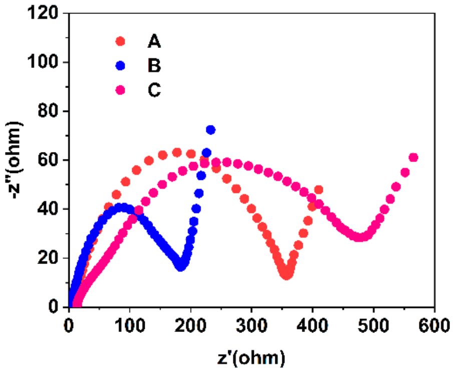

Figure S5. Electrochemical impedance diagram (Nyquist plots) of 3D-printed electrodes after deformation.

Table S1. Equivalent circuit fitting results after deformation of 3D printed electrode

Sample	A	B	C
$R_s(\Omega)$	6.306	2.668	9.895
$R_{ct}(\Omega)$	355.95	183.43	519.84
R_s increase rate vs. before deformation	109%	30%	139%
R_{ct} increase rate vs. before deformation	110%	30%	140%

The data clearly shows that Sample B has the lowest Rs (2.668 Ω) and Rct (183.43 Ω) after deformation, with the smallest increase rates (30% for both Rs and Rct), which further confirms its excellent structural stability and electrochemical performance under mechanical deformation. In contrast, Sample C has the highest Rs and Rct, along with the largest increase rates (139% and 140%, respectively), indicating that its structural damage during deformation significantly impairs its electrical conductivity and ion transport capability. Sample A shows intermediate performance between Sample B and Sample C, with moderate resistance values and increase rates.
